# Primate Torpor: Regulation of Stress-activated Protein Kinases During Daily Torpor in the Gray Mouse Lemur, *Microcebus murinus*

**DOI:** 10.1016/j.gpb.2015.03.002

**Published:** 2015-06-18

**Authors:** Kyle K. Biggar, Cheng-Wei Wu, Shannon N. Tessier, Jing Zhang, Fabien Pifferi, Martine Perret, Kenneth B. Storey

**Affiliations:** 1 Institute of Biochemistry and Department of Biology, Carleton University, Ottawa, ON K1S 5B6, Canada; 2 UMR 7179 Centre National de la Recherche Scientifique, Muséum National d′Histoire Naturelle, 91800 Brunoy, France; 3 Biochemistry Department, Schulich School of Medicine and Dentistry, Western University, London, ON N6A 5C1, Canada; 4 Department of Biology, Genetics Institute, University of Florida, Gainesville, FL 32611, USA; 5 Department of Surgery & Center for Engineering in Medicine, Massachusetts General Hospital & Harvard Medical School, Charlestown, MA 02129, USA; 6 Chemistry and Chemical Engineering Department, Royal Military College of Canada, Kingston, ON K7K 7B4, Canada

**Keywords:** Metabolic rate depression, Signal transduction, Mitogen activated protein kinase

## Abstract

Very few selected species of primates are known to be capable of entering torpor. This exciting discovery means that the ability to enter a natural state of dormancy is an ancestral trait among primates and, in phylogenetic terms, is very close to the human lineage. To explore the regulatory mechanisms that underlie primate torpor, we analyzed signal transduction cascades to discover those involved in coordinating tissue responses during torpor. The responses of mitogen-activated protein kinase (MAPK) family members to primate torpor were compared in six organs of control (aroused) versus torpid gray mouse lemurs, *Microcebus murinus.* The proteins examined include extracellular signal-regulated kinases (ERKs), c-jun NH_2_-terminal kinases (JNKs), MAPK kinase (MEK), and p38, in addition to stress-related proteins p53 and heat shock protein 27 (HSP27). The activation of specific MAPK signal transduction pathways may provide a mechanism to regulate the expression of torpor-responsive genes or the regulation of selected downstream cellular processes. In response to torpor, each MAPK subfamily responded differently during torpor and each showed organ-specific patterns of response. For example, skeletal muscle displayed elevated relative phosphorylation of ERK1/2 during torpor. Interestingly, adipose tissues showed the highest degree of MAPK activation. Brown adipose tissue displayed an activation of ERK1/2 and p38, whereas white adipose tissue showed activation of ERK1/2, p38, MEK, and JNK during torpor. Importantly, both adipose tissues possess specialized functions that are critical for torpor, with brown adipose required for non-shivering thermogenesis and white adipose utilized as the primary source of lipid fuel for torpor. Overall, these data indicate crucial roles of MAPKs in the regulation of primate organs during torpor.

## Introduction

The ability to generate internal heat and maintain a high body temperature (*T*_b_) has huge advantages for mammals, *e.g.*, supporting advanced cognitive capabilities, speed and agility, and ability to live in cold climates [1]. However, there are high energetic costs to a life as a warm-blooded mammal. Many small mammals live “right on the edge” with the amount of food that they eat each day barely sufficient to keep them alive and warm until morning [2]. As a result, added stresses on the animal, particularly seasonal shortages of food/water and/or extreme environmental temperatures can be lethal. The solution for many small mammals is to temporarily lower their energy needs by regulating a strong suppression of their metabolic rate, causing *T*_b_ to fall, and enter either short-term daily torpor or long-term (days or even weeks) continuous hibernation [3–5].

Although alien to humans, daily torpor and hibernation occur in multiple mammalian groups including monotremes, marsupials, rodents, bats, and bears [4]. Various ground squirrel, bat, hamster, and mouse species have been main models for most of the lab-based studies of the biochemical and genetic control of the phenomena [3–5]. It is now known that torpor also occurs in a few species of primates – specifically, some lemurs that are native to Madagascar [2,6–8]. In phylogenetic terms, this indicates that the ability to enter torpor is an ancestral trait of the primate lineage, occurring among species that are very close to the human line [9].

Mouse lemurs of the *Microcebus* genus are the smallest primates in the world but among these, the gray mouse lemur, *Microcebus murinus*, is the largest (weighing around 85–110 g) [2]. Mouse lemurs are nocturnal and sleep in tree holes during the day. They are found along the entire west coast of Madagascar with other populations in the north-central and south-eastern regions of the country. This land is lush and wet during the summer but cool and dry in the winter [10]. To deal with periodic food shortages, limited water supply, and cool winter temperatures, mouse lemurs employ hypometabolism [7,8,10]. Daily torpor occurs frequently in mouse lemur species and multi-day hibernation has also been reported for both *M. murinus* and *Microcebus griseorufus* [8]. Environmental factors such as photoperiod, ambient temperature, and food availability are each involved in shaping the biological rhythms of these animals that are characterized by a winter resting period, an active summer breeding season, and an autumn fattening stage [2]. Although several studies have documented the physiological responses of mouse lemur torpor/hibernation [8], this study and the others in this series are the first to explore some of the molecular mechanisms that regulate the phenomena.

Many studies of small mammal torpor and hibernation have discovered compelling commonalities. A prominent theme is a strong global suppression of metabolic rate, involving a regulated and coordinated reduction in all metabolic processes [3,11]. Moreover, fine cellular controls are needed to selectively modulate gene expression and direct specific cellular responses to meet the unique needs of individual organs [3,11]. Established models of mammalian hibernation are typically coincident with low *T*_b_ values [5]. By strongly suppressing energy-expensive cell functions and letting Tb values cool to ambient (sometimes as low as 0–5 °C), metabolic rate can often be reduced to <5% of normal resting values in euthermia [1]. While hibernation provides an eloquent solution to seasonal shortages; it is often difficult to distinguish between the specific molecular adaptations necessary for metabolic rate depression from those that contribute to surviving cold *T*_b_. This has been a significant area of controversy in hibernation research [12]. Interestingly, summer-active hibernating species are as susceptible to metabolic damage from hypothermia or hypoxia insults as are non-hibernating mammals, but during the winter they can easily transition into torpor, letting *T*_b_ fall to near 0 °C and displaying substantially-enhanced hypoxia/ischemia tolerance [11,12]. The lemur model is extremely attractive for studies of mammalian hypometabolism and has several advantages as a model: (a) as primates, these animals are the most closely-related species to human that exhibit natural hypometabolism, and (b) they enter torpor at high ambient temperatures so they show a “pure” form of hypometabolism that is not confounded by the additional biochemical adaptations needed by most species to adjust enzymes/proteins for low temperature function.

Studies from a range of animal models with various tolerances to different environmental stresses (freezing, anoxia, low pH, dehydration, *etc*.) have shown that global metabolic rate suppression, mediated via reversible protein phosphorylation (RPP), is crucial for survival [3]. RPP can produce major changes in the activity states of many enzymes and functional proteins, often providing on/off control. Apart from direct regulation of functional proteins, RPP is also responsible for the detection of extracellular stimuli and their propagation via intracellular signal transduction networks. The use of RPP provides a fast and coordinated mechanism to regulate the function of a wide number of cellular processes that can also be rapidly reversed once the stress is removed [13]. In particular, important targets for RPP control include proteins involved in catabolic pathways that regulate ATP supply, and proteins that regulate major ATP-consuming cell processes such as transmembrane ion transport, gene transcription, and protein translation [3,14]. Given the extensive research that RPP has received, it has been well-established that RPP is a central mechanism in the coordination of cellular functions throughout bouts of torpor/hibernation and arousal in hibernating species, just as it does in many other systems of natural hypometabolism [15,16].

In light of an overall suppression of gene transcription and protein translation, expression of a small number of genes that are necessary for survival is upregulated in the hypometabolic animal [3]. The identification and characterization of these upregulated genes is particularly important as they provide insight into what cellular functions are important for long-term survival [17,18]. To date, these important cellular functions have been shown to include protein chaperones, proteins involved in reorganization of fuel metabolism, antioxidant defense, muscle restructuring, system regulators (growth, cell cycle, apoptosis, atrophy), and proteins that support non-shivering thermogenesis [16,19–21].

It has been well documented that mitogen-activated protein kinase (MAPK) signaling pathways are crucial in the regulation of the cellular stress response, and several studies have documented their role in hibernation at low body temperatures [15,22–30]. In the lemur, the activation of selected MAPK signaling pathways may provide a rapid response mechanism for stress-responsive gene expression, contributing to the control of torpor entry/exit. The present study examined the protein expression and phosphorylated state of extracellular signal-regulated kinase 1/2 (ERK1/2), MAPK kinase (MEK), c-jun NH_2_-terminal kinase (JNK), and p38, as well as the downstream targets heat shock protein 27 (HSP27) and p53, in different tissues of lemurs by comparing torpid animals with control aroused animals. By utilizing an animal that can enter hypometabolism without the confounding effects of low *T*_b_, the majority of cellular reorganization seen during torpor at warm *T*_b_ values can be attributed to active induction of metabolic rate depression.

## Results

**Figure 1:**
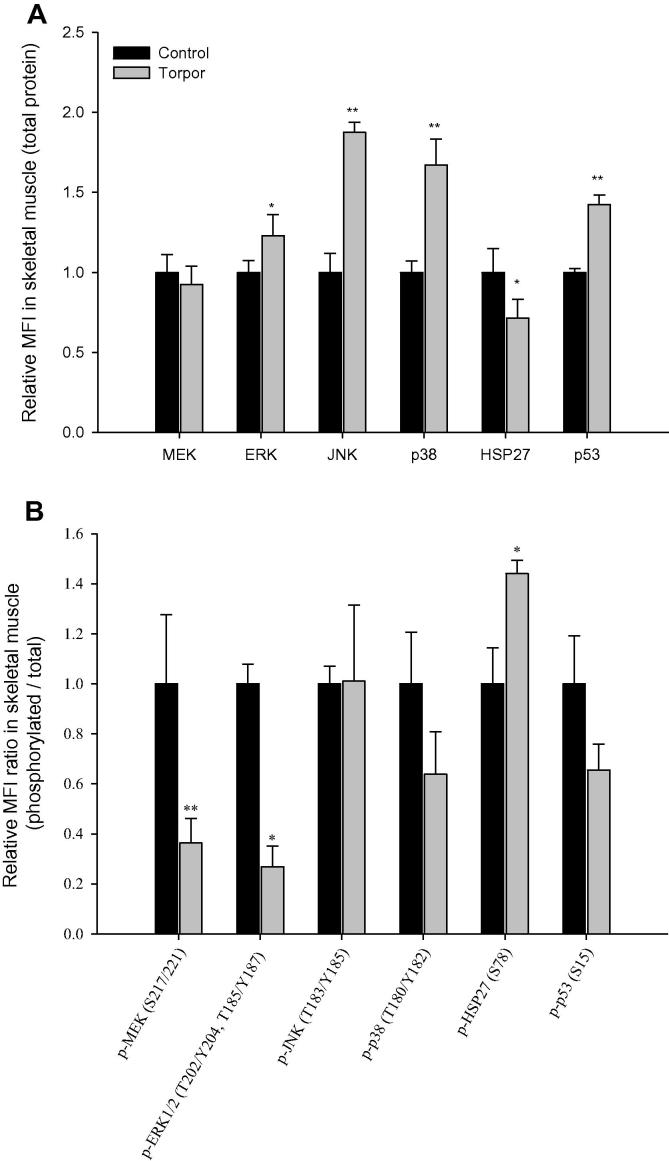
**Expression and phosphorylation of MAPK kinases and targets in skeletal muscle of gray mouse lemurs** **A.** Relative total protein levels of ERK, MEK, JNK, p38, HSP27, and p53. **B.** Relative ratio of phosphorylation levels (relative phosphorylation/relative total protein expression) of p-ERK1/2 (Thr202/Tyr204, Thr185/Tyr187), p-MEK (Ser217/221), p-JNK (Thr183/Tyr185), p-p38 (Thr180/Tyr182), p-HSP27 (Ser78), and p-p53 (Ser15). All data were obtained by multiplex analysis using a Luminex 100 instrument and analyzed with Milliplex analyst software. Shown are histograms of mean of median fluorescent intensity (MFI) of immune-reactive multiplex beads (mean ± SEM), *n* = 4 independent samples from different animals from control (aroused) and torpid experimental conditions. Significant differences in torpid animals in comparison to controls are denoted with ^*^(*P* < 0.05) and ^**^(*P* < 0.01), respectively (two-tailed Student *t*-test).

**Figure 2:**
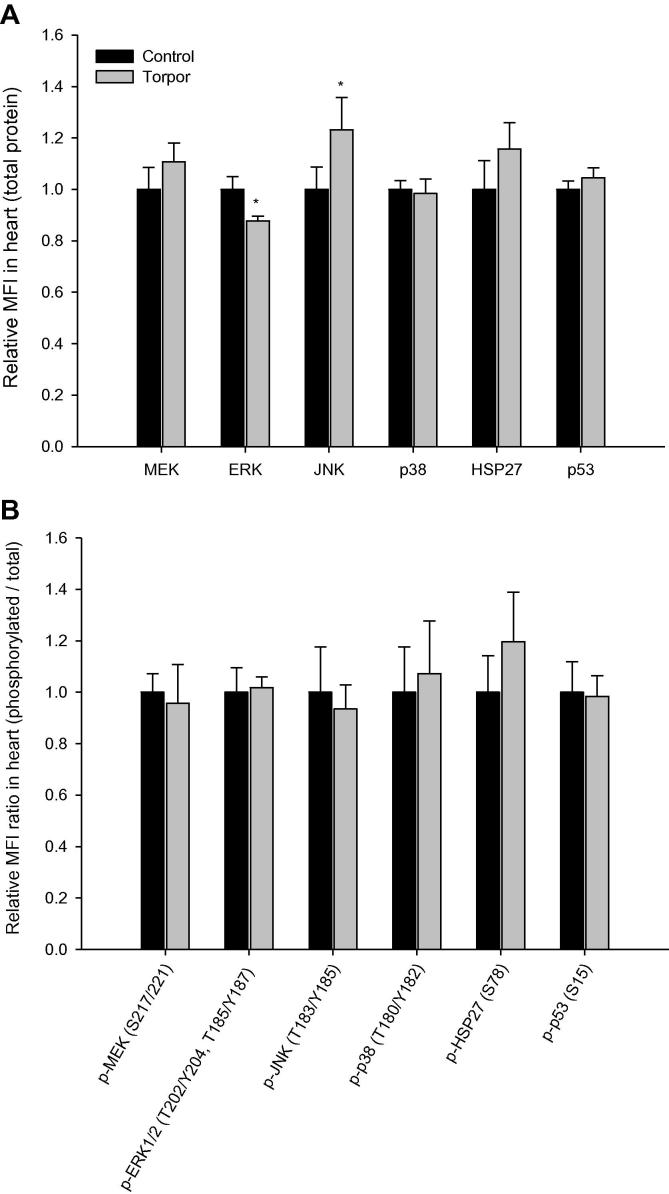
**Expression and phosphorylation of MAPK kinases and targets in the heart of gray mouse lemurs** Significant differences in torpid animals in comparison to controls are denoted with ^*^(*P* < 0.05) and ^**^(*P* < 0.01), respectively (two-tailed Student *t*-test).

Using custom multiplex panels, which were validated to cross-react in both primate and rodent species (Bio-Rad, Hercules, CA), relative changes in total and phosphoprotein expression of ERK, MEK, JNK, p38, HSP27, and p53 were assessed in the skeletal muscle, heart, liver, kidney, brown adipose tissue (BAT), and white adipose tissue (WAT) by comparing control (aroused) and torpid lemurs. In skeletal muscle, relative protein levels of ERK, JNK, p38, and p53 were significantly increased in torpid animals, which are 1.2 ± 0.1 (*P* < 0.05), 1.9 ± 0.1 (*P* < 0.01), 1.7 ± 0.2 (*P* < 0.01), and 1.4 ± 0.1 (*P* < 0.01) fold of those in controls, respectively (Figure 1**A**). In contrast, the relative expression of HSP27 in skeletal muscle decreased significantly, being 72 ± 11% of the control value (*P* < 0.05). The phosphorylation of three targets in skeletal muscle also changed when comparing control and torpor conditions. During torpor, the ratio (*i.e.*, relative phosphorylation/relative total expression) of phosphorylation for HSP27 (Ser78) was significantly increased, being 1.4 ± 0.1 fold of that in control animals (*P* < 0.05), whereas the relative phosphorylation ratios of ERK1/2 (Thr202/Tyr204, Thr185/Tyr187) and MEK (Ser217/221) phosphorylation were lower, 36 ± 10% (*P* < 0.01) and 27 ± 8% (*P* < 0.05) of those in control animals, respectively (Figure 1**B**). In the heart tissue (Figure 2**A**), ERK levels in the heart during torpor were 87 ± 2% of the control (aroused) value (*P* < 0.05), whereas JNK levels during torpor were 1.2 ± 0.1 fold of controls (*P* < 0.01), with no appreciable changes observed in other targets. Assessment of the relative phosphorylation ratios of the targets in the heart showed no significant changes (Figure 2**B**).

**Figure 3:**
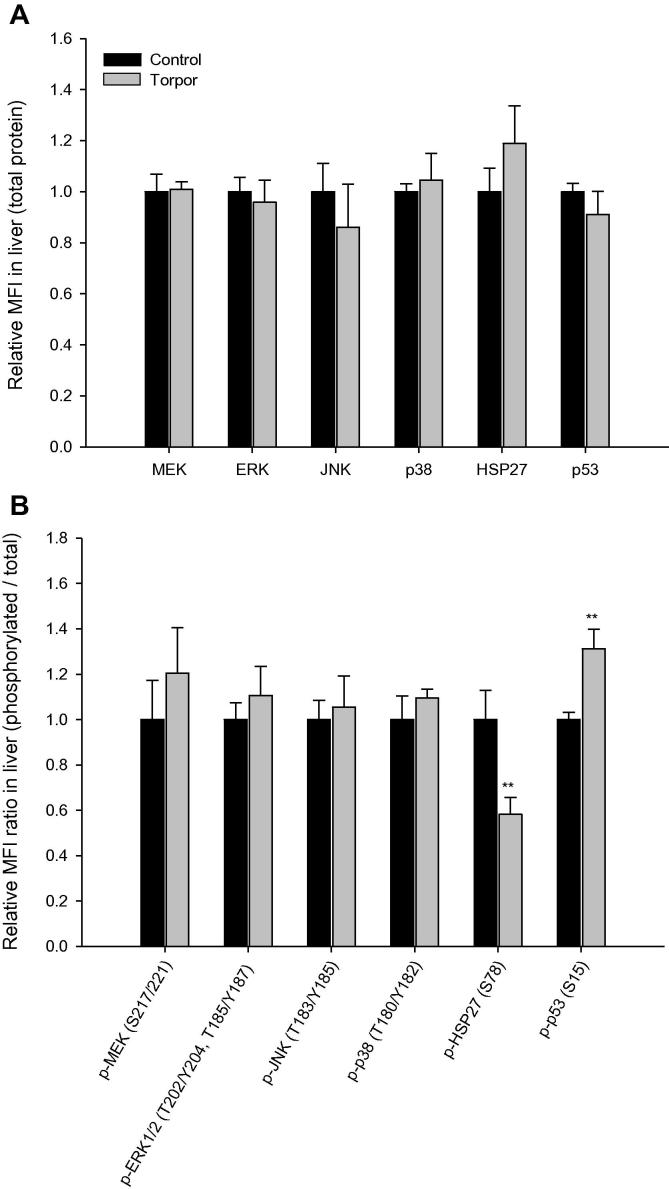
**Expression and phosphorylation of MAPK kinases and targets in the liver of gray mouse lemurs** Significant differences in torpid animals in comparison to controls are denoted with ^*^(*P* < 0.05) and ^**^(*P* < 0.01), respectively (two-tailed Student *t*-test).

**Figure 4:**
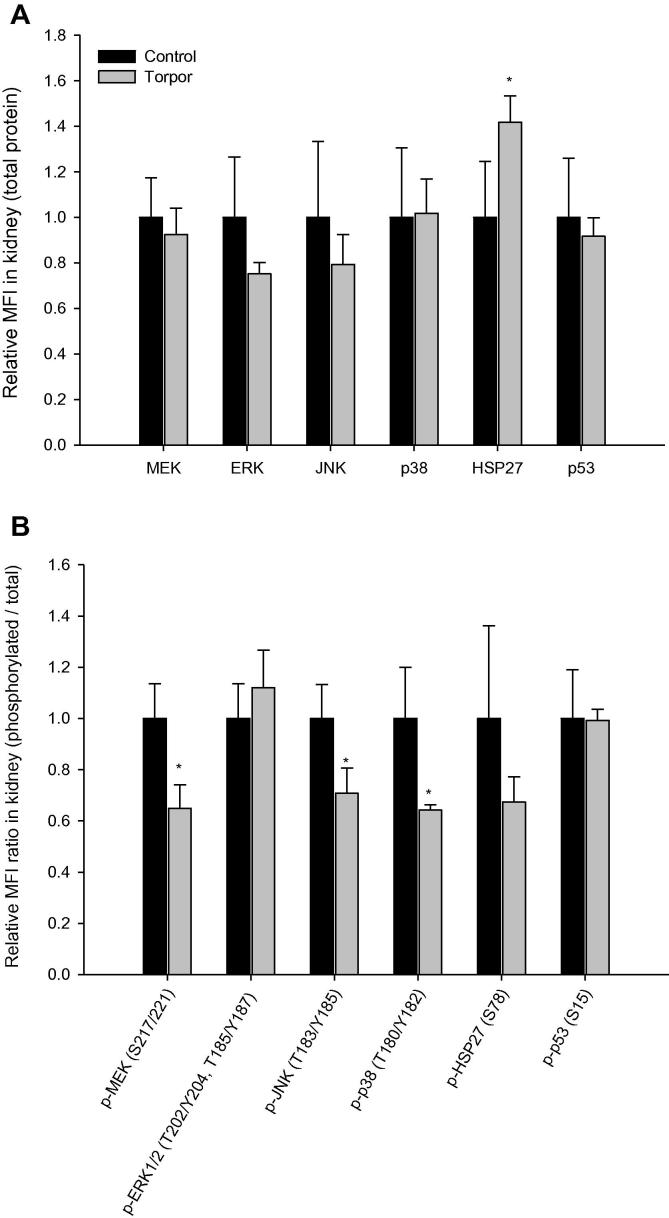
**Expression and phosphorylation of MAPK kinases and targets in kidney of gray mouse lemurs** Significant differences in torpid animals in comparison to controls are denoted with ^*^(*P* < 0.05) and ^**^(*P* < 0.01), respectively (two-tailed Student *t*-test).

In the liver, total protein levels of all six targets were unchanged in response to torpor (Figure 3**A**). However, the ratio of relative phosphorylated p53 (Ser15) was significantly higher (1.3 ± 0.09 fold, *P* < 0.01) during torpor, whereas relative HSP27 (Ser78) phosphorylation was lower (58 ± 8% of the control value; *P* < 0.01) (Figure 3**B**). In kidney, the relative protein expression of HSP27 was elevated (1.4 ± 0.1-fold) during daily torpor (*P* < 0.05), the only one of the targets to show a significant change (Figure 4**A**). However, the relative ratios of phosphorylation for three targets were reduced significantly and substantially in kidney (Figure 4**B**) during torpor. The relative phosphorylation of MEK (Ser217/221), JNK (Thr183/Tyr185), and p38 (Thr180/Tyr182) decreased to 65 ± 9%, 71 ± 10%, and 64 ± 2% of control (aroused) values, respectively (*P* < 0.05).

**Figure 5:**
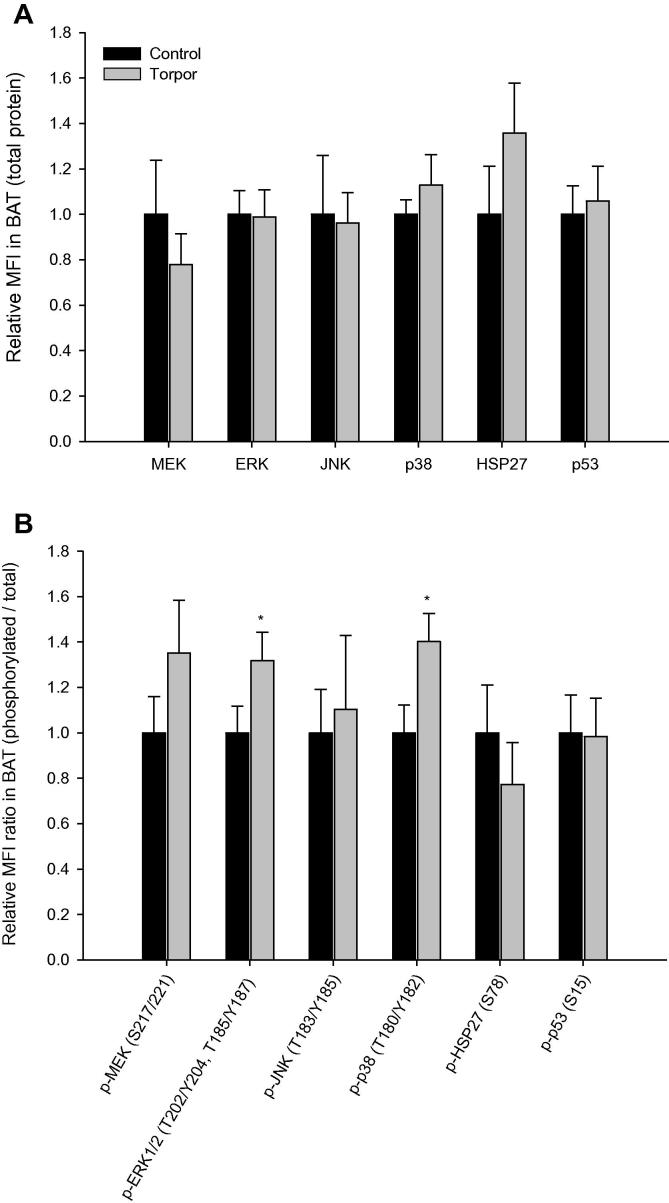
**Expression and phosphorylation of MAPK kinases and targets in brown adipose tissue of gray mouse lemurs** Significant differences in torpid animals in comparison to controls are denoted with ^*^(*P* < 0.05) and ^**^(*P* < 0.01), respectively (two-tailed Student *t*-test).

**Figure 6:**
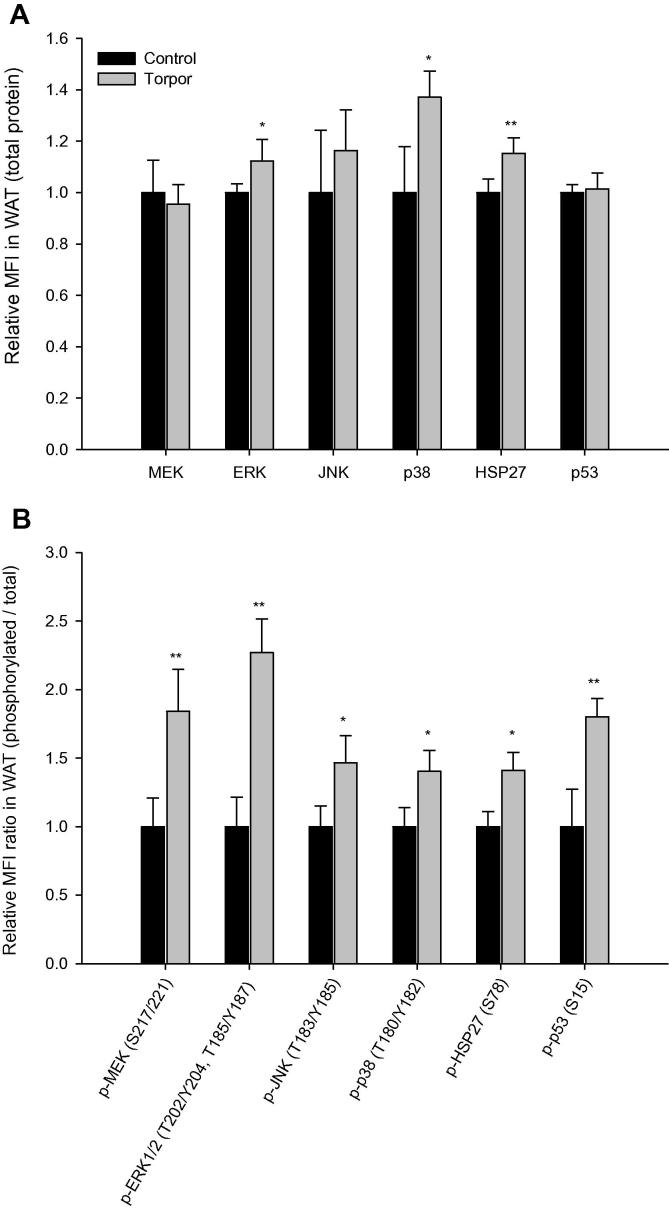
**Expression and phosphorylation of MAPK kinases and targets in white adipose tissue of gray mouse lemurs** Significant differences in torpid animals in comparison to controls are denoted with ^*^(*P* < 0.05) and ^**^(*P* < 0.01), respectively (two-tailed Student *t*-test).

No significant changes were observed for any of the six total protein targets analyzed in BAT during torpor (Figure 5**A**), but phosphorylation of ERK1/2 (Thr202/Tyr204, Thr185/Tyr187) and p38 (Thr180/Tyr182) were significantly elevated, being 1.3 ± 0.1 and 1.4 ± 0.1 fold of that in control animals, respectively (Figure 5**B**; *P* < 0.05). Data for WAT showed that total protein levels of ERK, p38, and HSP27 were significantly increased during torpor, which are 1.1 ± 0.1 (*P* < 0.05), 1.4 ± 0.1 (*P* < 0.05), and 1.1 ± 0.1 (*P* < 0.01) fold of those in control (aroused) animals, respectively (Figure 6**A**). Furthermore, in WAT of torpid lemurs, all six targets exhibited significant elevation in phosphorylation as compared to control animals, with fold changes of 1.8 ± 0.3 (MEK Ser217/221; *P* < 0.01), 2.3 ± 0.2 (ERK1/2 Thr202/Tyr204, Thr185/Tyr187; *P* < 0.01), 1.5 ± 0.2 (JNK Thr183/Tyr185; *P* < 0.05), 1.4 ± 0.2 (p38 Thr180/Tyr182; *P* < 0.01), 1.4 ± 0.1 (HSP27 Ser78; *P* < 0.05), and 1.8 ± 0.1 (p53 Ser15; *P* < 0.01), in relative to total protein expression (Figure 6**B**).

## Discussion

Mammals have the ability to maintain a constant high core *T*_b_ over a wide range of ambient temperatures. However, during periods of food restriction, often associated with cold or dry conditions, the high cost of metabolic heat production can be detrimental to survival. Accordingly, some mammals display significant heterothermy and/or use torpor as means to conserve energy during periods of severe environmental stress [1,3,11,12]. Torpor in heterothermic endotherms is characterized by a controlled reduction of metabolic rate that then results in a drop in *T*_b_, often to near ambient temperature, as well as a suppression of multiple physiological functions (*e.g.*, heart rate and breathing rate) [1,11,12]. Low temperatures often confound experiments aimed at studying the regulation of torpor in classic hibernators [12,31–33]. However, torpor/hibernation in the tropics or subtropics provides researchers with models of mammalian hypometabolism, which are much more “pure” in the sense that the effects of adaptations to low body temperatures can be factored out of the analysis. Indeed, *T*_b_ at the nadir of daily torpor in the mouse lemurs used in the present study was only 30–33 °C. Hence, studies of torpor in the gray mouse lemur offer the option to explore the regulation of a relatively pure reduction in metabolic rate, without the confounding influence of low temperature.

To date, the majority of research on mammalian torpor and hibernation has focused on species from seasonally-cold environments such as bats and a variety of rodents including ground squirrels, hamsters, chipmunks, and marmots, whose seasonal heterothermy is necessitated by the subzero winter temperatures that must be endured [16,34,35]. Although lemurs live in tropical Madagascar, they experience similar periods of heterothermic torpor that allow them to deal with the dry cool season when food is scare. This shows that torpor/hibernation is not just a temperature-driven phenomenon. Rather, induction of a heterothermic phenotype is more strongly linked to the seasonal availability of nutrients [33], as is estivation, another form of hypometabolism that is typically associated with hot, arid conditions that also impact food availability [36]. As yet, there have been few studies examining the molecular mechanisms of primate torpor. The present study focused on the MAPK cascades that are major cell signaling pathways mediating environmental stress signals with primary effects on gene expression. The expression and relative phosphorylation status of three MAPK cascades (ERK1/2, p38, and JNK) were characterized in six tissues of gray mouse lemurs comparing aroused and torpid animals. Interestingly, we observed unique patterns of tissue-specific response in all three MAPK cascades, when the torpor-responsive MAPK expression in the lemur was compared to expression in models of mammalian hibernation at low body temperatures.

The ERK signaling pathway is initiated by the activation of G-proteins, which leads to the sequential phosphorylation and activation a three-tiered cascade involving a MAPK kinase kinase, a MAPK kinase and finally the MAPK. For the ERK cascade the three kinases are RAF kinase (MAPKKK) → MEK (MAPKK) → ERK (MAPK). Upon activation by phosphorylation at Thr202 and Tyr204, ERK1 and ERK2 function as potent Ser/Thr kinases that phosphorylate a wide array of downstream targets. To date, approximately 160 different ERK substrates have been discovered with the majority of downstream proteins involved in regulating cellular proliferation, growth, and survival [37,38]. In the lemur, both MEK and ERK1/2 displayed unique patterns across six tissues during torpor. Despite an increase in ERK total protein in skeletal muscle, a strong reduction in the relative levels of phosphorylation for ERK1/2 and MEK was evident during torpor (Figure 1). Such reduction might be attributed to a decrease in the level of mitogenic stimuli during torpor; suggesting that cell growth and proliferation are suppressed in skeletal muscle during torpor. In other tissues, a moderate reduction of total ERK1/2 protein level was observed in the heart whereas a reduction of relative phosphorylated MEK occurred in kidney during torpor.

Interestingly, both BAT and WAT showed increases in relative ERK1/2 phosphorylation during torpor, indicative of increased ERK action. The thermogenic BAT is the main source of heat for rewarming the body during the arousal phase of torpor/hibernation, whereas WAT is the primary metabolic fuel storage organ [39]. Both showed a significant increase in ERK1/2 phosphorylation in torpor (Figures 5 and 6B) and WAT also showed a small increase in ERK1/2 protein levels and relative phosphorylation of MEK. Thus, as opposed to the trend observed in skeletal muscle, WAT showed a dramatic increase in ERK signaling, suggesting a possible increase of cellular functions such as proliferation and growth in this tissue during torpor. Studies have recently shown that wintering lemurs spare the consumption of body protein mass during periods of food shortage, indicating a reliance on lipids, mobilized from WAT, as the primary source of metabolic fuel in torpor [2]. This seasonal shift in oxidative metabolic fuel results in a 6-fold increase of fat mass during winter preparation in lemurs as compared to summer animals [2]. Fat storage and reliance on lipids as the main metabolic fuel is well-known for other models of mammalian torpor/hibernation. For example, rodent hibernators display a respiratory quotient of 0.7 during torpor, a value indicative of nearly 100% fatty acid oxidation [40]. As well, key enzymes of carbohydrate catabolism such as pyruvate dehydrogenase and glycogen phosphorylase are suppressed in hibernating ground squirrels, indicating conservation of carbohydrate reserves [41].

Like ERK1/2, both JNK and p38 are activated via phosphorylation by a direct upstream MAPK kinase and are primarily involved in stress-activated cellular responses. Although both JNK and p38 cascades can be activated through similar stimuli, each cascade elicits distinct downstream responses [42–46]. JNK is involved in pro-apoptotic signaling through phosphorylation of p53 at Ser15, which is critical in stabilization of p53 [43,44]. An upregulation of JNK protein levels was evident only in the two muscle types (cardiac and mixed skeletal) during torpor in lemurs whereas relative phosphorylation of JNK occurred only in WAT (Figures 1–6). Since the functional role of JNK phosphorylation has been linked to numerous downstream processes, it is often hard to predict its primary function when activated. Nonetheless, an increase in p53 phosphorylation at Ser15 was correlated with JNK phosphorylation in WAT. Whereas JNK is a known regulator of p53 activity, the broad spectrum of JNK downstream effectors and variety of kinases upstream of p53 provides possible explanations for the discord between JNK and p53 activation in other tissues. For example, ataxia telangiectasia mutated (ATM) and ATM- and Rad3-related (ATR) kinases both phosphorylate p53 at Ser15 in response to DNA damage [47,48]. In summary, the lack of correlation between JNK and p53 phosphorylation pattern in selected tissues suggests that the regulation of JNK signaling could play a minor role in regulation of apoptosis (mediated by p53) during torpor, and the activation of JNK in selected tissues functions to activate other downstream targets, which have yet to be identified in the lemur.

The third subfamily of MAP kinases, p38, is also activated through similar extracellular stimuli as JNK and, as such, it was predicted that the regulatory pattern of p38 would be similar to that observed for JNK during torpor. Total p38 protein expression was upregulated in skeletal muscle and WAT; however, active phosphorylated p38 (Thr108/Tyr182) increased in BAT, and WAT, while decreasing in the kidney (Figures 1–6). As mentioned, distinct downstream phosphorylation targets for p38 and JNK have been each identified. For example, HSP27, a chaperone protein that is activated during periods of cellular stress, lies downstream of p38 [49]. Our findings show that although tissue-specific patterns of HSP27 expression are evident during torpor, only WAT displayed a correlation between HSP27 and p38 phosphorylation. Similar to p53, HSP27 can be phosphorylated by a number of different upstream kinases, including p90 ribosomal S6 kinase (RSK) and protein kinase C (PKC) [50,51]. Although the selected JNK/p38 downstream targets measured in this study did not uncover evidence for pathway activation, the increased phosphorylation of JNK and p38 (which is necessary for kinase activity) observed in torpor suggests the presence of stress-activated stimuli in selected tissues of the lemur during torpor.

When comparing the regulation pattern of the three MAP kinase subgroups, no uniform pattern was discerned between tissues during torpor. In the skeletal muscle, decrease in relative phosphorylation of ERK and its upstream kinase MEK was most evident. By contrast, kidney showed no changes in total protein levels of any of the MAPKs (or MEK) but differed in relative phosphorylation of the kinases, displaying a notable decrease in phosphorylated JNK and p38 (as well as MEK). The most interesting findings of this study were the patterns observed in the adipose tissues during torpor. Both BAT and WAT showed significant increases in relative ERK and p38 phosphorylation, with WAT exhibiting an increased phosphorylation of all three MAP kinases (and MEK) during torpor. As mentioned earlier, both adipose tissues possess specialized functions that are critical for torpor, with BAT required for non-shivering thermogenesis and WAT as the primary source of metabolic fuel [52,53].

The relative changes in both total and phosphorylated ERK1/2, JNK, MEK and p38 kinase expression observed in the organs of the hibernating gray mouse lemur, *M. murinus*, suggest important cellular roles for MAPK signaling in adapting the animal for torpor. The activation of these selected signal transduction pathways may influence the expression of a panel of torpor-responsive genes, which are critical to the regulation of distinct cellular processes that are necessary for survival in the metabolically-depressed state. Hence, the role of MAPK signaling may involve the execution of unique, tissue-specific survival programs that support torpor in this primate model. Given current efforts to sequence the genome of *M. murinus*, it is possible that future proteomic analysis of primate torpor will be able to identify torpor-responsive signaling pathways unique to either daily torpor or seasonal hibernation.

## Materials and methods

### Animals

Gray mouse lemurs (*M. murinus*) used for this study consisted of 8 adult females, 2–3 years of age (mean body mass 106.3 ± 15.5 g), that were born in the authorized breeding colony at the National Museum of Natural History of France (Brunoy, France; European Institution Agreement # E91-114-1). Standard procedures for animal holding, experimentation and sampling were conducted by Dr. Martine Perret and the Adaptive Mechanisms and Evolution team (MECADEV-Mecanismes Adaptatifs et Evolution, Department of Ecology and Management of Biodiversity) as previously described [2,54]. In the breeding colony, mouse lemurs were exposed to a photoperiod regime that parallels the alternating 6 month winter short days (10 h light/day) and 6 month summer long days (14 h light/day) of Madagascar.

All animals in the present study were under winter short day conditions for at least 6 weeks before being transferred to individual cages (0.5 × 0.3 × 0.3 m) that were visually separated from each other and contained nest-boxes and branches. Cages were housed in a climate chamber where all mouse lemurs were maintained under short day conditions at a thermoneutral ambient temperature (24–25 °C) with a constant relative humidity (55%). *T*_b_ and locomotor activity were recorded with internal thermosensitive radio-transmitters (TA10TA-F20, 3.2 g, Data Sciences International, St Paul, MN) and receiver boards (RPC-1, Data Sciences International) placed in each cage. Body temperature was recorded for 10 s every 5 min whereas locomotor activity was continuously recorded.

Mouse lemurs were separated into two experimental groups (*n* = 4 each for control and torpid groups). Lemurs in the torpor group were exposed to a calorie-restricted diet for 5 days (60% of the control diet; 86 × 10^−3^ J/day versus 144 × 10^−3^ J/day for controls) to enhance the depth of their torpor bouts. Control animals were also capable of entering torpor but were fed *ad libitum*. *T*_b_ and locomotion were monitored and used to determine the state of torpor, which was assessed as a continuously low *T*_b_ (with no evidence of animal activity). Control animals were euthanized at the end of a daily torpor bout (*i.e.*, after spontaneous rewarming to 35−36 °C), whereas torpid mouse lemurs were euthanized during a torpor bout (when *T*_b_ was at its minimum). For the animals in the present study, the nadir *T*_b_ was 30.8 ± 1.6 °C (range 30–33 °C). Animals were euthanized by decapitation following approved protocols used by the MECADEV team. Samples of selected tissues were rapidly excised, immediately frozen in liquid nitrogen and subsequently air freighted to Carleton University on dry ice where they were stored continuously at −80 °C until use. All animal experiments were performed in accordance with the Principles of Laboratory Animal Care (National Institutes of Health publication 86–23, revised 1985) and the European Communities Council Directive (86/609/EEC). All experiments were conducted under personal license (license No. C91-563) and the Internal Review Board of the UMR 7179. In accordance with the recommendations of the Weatherall report, “The use of non-human primates in research”, special attention was paid to the welfare of animals during this work. All efforts were made to minimize nociception. For transport, all tissues were logged as per Convention on International Trade in Endangered Species of Wild Flora and Fauna (CITES) regulations under CITES export permit No. FR1009118231-E and CITES import permit No. 10cA02291/QWH.

### Multiplex analysis

Two custom multiplex assay panels were prepared by Bio-Rad (Hercules, CA) and used to measure the total protein levels of ERK1/2, MEK1, JNK, p38, HSP27, and p53 as well as their phosphorylated forms including p-ERK1/2 (Thr202/Tyr204, Thr185/Tyr187), p-MEK (Ser 217/221), p-JNK (Thr183/Tyr185), p-p38 (Thr180/Tyr182), p-HSP27 (Ser 78), and p-p53 (Ser15). All antibodies assay panels used were previously demonstrated by the manufacturer to cross-react with both primate and rodent species (*i.e.*, human, mouse, and rat). Lysis, assay, wash and resuspension buffers were all supplied by Bio-Rad (Cat. No. 171-304011).

Extracts of frozen tissue samples were prepared as per manufacturer’s instructions. Briefly, ∼50 mg samples were weighed and homogenized in lysis buffer with a Dounce homogenizer with 2 mM phenylmethylsulfonyl fluoride (PMSF) added. Samples were then frozen at −80 °C and thawed at room temperature. After another freeze-thaw treatment at −20 °C, homogenates were centrifuged at 4000 × *g* for 4 min and supernatants were collected as total soluble protein lysates. Protein concentration of the lysates was determined using the Bradford assay with the Bio-Rad prepared reagent and then further diluted to an appropriate concentration using assay buffer.

Premixed coupled beads for all the protein targets were diluted by mixing with wash buffer. A 96-well filter microplate was then prepared by adding wash buffer to the desired number of wells and drawing the buffer through the plate by vacuum. A 50 μl aliquot of diluted coupled beads was then added to each well. After washing twice, 50 μl of sample lysate (protein concentration 500 μg/ml) was added to the wells and incubated overnight on a shaker. The detection antibodies (25 μl) were then added to each well and incubated for 30 min. The antibody solution was then drawn through the wells by vacuum pressure. After washing, 50 μl of 1 × streptavidin-PE (diluted in wash buffer) was added to each well and incubated for 10 min. Wells were then vacuumed and washed with 100 μl of resuspension buffer for a total of three washes. After washing, 125 μl of re-suspension buffer was added into each well and then data acquisition was carried out on a Luminex 100 instrument (Luminex, Austin, TX) with Milliplex analyst software (Millipore, Billerica, MA).

### Statistical analysis

Data was collected as median fluorescent intensity (MFI) of the immunoreactive multiplex beads detected by the Luminex 100 instrument. All numerical data are expressed as means ± SEM (*n* = 4). Statistical analysis was performed using SigmaPlot (v.11) software using a two-tailed Student’s *t*-test. Differences were considered significant at *P* < 0.05 or *P* < 0.01.

## Authors’ contributions

All authors contributed to the conception and design of the project and to the editing of the manuscript. MP and FP carried out the animal experiments. KKB, CWW, SNT, and JZ conducted biochemical assays. Data analysis and assembly of the draft manuscript was carried out by KBS, KKB and CWW. All authors read and approved the final manuscript.

## Competing interests

The authors have declared no competing interests.

## References

[1] Wang L.C.H., Lee T.F. (2011). Torpor and hibernation in mammals: metabolic, physiological, and biochemical adaptations. Comp Physiol.

[2] Giroud S., Blanc S., Aujard F., Bertrand F., Gilbert C., Perret M. (2008). Chronic food shortage and seasonal modulation of daily torpor and locomotor activity in the grey mouse lemur (*Microcebus murinus*). Am J Physiol.

[3] Storey K.B., Storey J.M. (2004). Metabolic rate depression in animals: transcriptional and translational controls. Biol Rev Camb Philos Soc.

[4] Storey K.B. (2009). Out cold: biochemical regulation of mammalian hibernation—a mini-review. Gerontology.

[5] Overton J.M., Williams T.D. (2004). Behavioral and physiologic responses to caloric restriction in mice. Physiol Behav.

[6] Bourliere F., Petter-Rousseaux A. (1966). Probable existence of a seasonal metabolic rhythm in Cheirogaleinae (Lemuroidea). Folia Primatol.

[7] Dausmann K.H., Glos J., Ganzhorn J.U., Heldmaier G. (2004). Hibernation in a tropical primate. Nature.

[8] Schmid J., Ganzhorn J.U. (2009). Optional strategies for reduced metabolism in gray mouse lemurs. Naturwissenschaften.

[9] Staples J.F. (2014). Metabolic suppression in mammalian hibernation: the role of mitochondria. J Exp Biol.

[10] Dausmann K.H., Glos J., Ganzhorn J.U., Heldmaier G. (2005). Hibernation in the tropics: lessons from a primate. J Comp Physiol B.

[11] Storey K.B., Storey J.M. (2007). Putting life on ‘pause’–molecular regulation of hypometabolism. J Exp Biol.

[12] Geiser F. (2004). Metabolic rate and body temperature reduction during hibernation and daily torpor. Annu Rev Physiol.

[13] Storey K.B. (1997). Metabolic regulation in mammalian hibernation: enzymes and protein adaptations. Comp Biochem Physiol A.

[14] MacDonald J.A., Storey K.B. (1999). Regulation of ground squirrel Na^+^K^+^-ATPase activity by reversible phosphorylation during hibernation. Biochem Biophys Res Commun.

[15] MacDonald J.A., Storey K.B. (2005). Mitogen-activated protein kinases and selected downstream targets display organ specific responses in the hibernating ground squirrel. Int J Biochem Cell Biol.

[16] Wu C.W., Storey K.B. (2012). Regulation of the mTOR signaling network in hibernating ground thirteen-lined ground squirrels. J Exp Biol.

[17] Eddy S.F., Storey K.B. (2002). Dynamic use of cDNA arrays: heterologous probing for gene discovery and exploration of organismal adaptation to environmental stress. Cell Mol Res Stress.

[18] Yan J., Barnes B.M., Kohl F., Marr T.G. (2007). Modulation of gene expression in hibernating arctic ground squirrels. Physiol Genom.

[19] Eddy S.F., Morin P., Storey K.B. (2005). Cloning and expression of PPARγ and PGC-1α from the hibernating ground squirrel, *Spermophilus tridecemlineatus*. Mol Cell Biochem.

[20] Morin P., Storey K.B. (2005). Cloning and expression of hypoxia-inducible factor 1α from the hibernating ground squirrel, *Spermophilus tridecemlineatus*. Biochim Biophys Acta.

[21] Tessier S.N., Storey K.B. (2010). Expression of myocyte enhancer factor-2 and downstream genes in ground squirrel skeletal muscle during hibernation. Mol Cell Biochem.

[22] Zhu X., Smith M.A., Perry G., Wang Y., Ross A.P., Zhao H.W. (2005). MAPKS are differentially modulated in artic ground squirrels during hibernation. J Neurosci Res.

[23] Seger R., Krebs E.G. (1995). The MAPK signaling cascade. FASEB J.

[24] Rouse J., Cohen P., Trigon S., Morange M., Alonso-Llamazares A., Zamanillo D. (1994). A novel kinase cascade triggered by stress and heat shock that stimulates MAPKAP kinase-2 and phosphorylation of the small heat shock proteins. Cell.

[25] Han J., Lee J.D., Bibbs L., Ulevitch R.J. (1994). A MAP kinase targeted by endotoxin and hyperosmolarity in mammalian cells. Science.

[26] Freshney N.W., Rawlinson L., Guesdon F., Jones E., Cowley S., Hsuan J. (1994). Interleukin-1 activates a novel protein kinase cascade that results in the phosphorylation of Hsp27. Cell.

[27] Wortzel I., Seger R. (2011). The ERK cascade: distinct functions within various subcellular organelles. Genes Cancer.

[28] Payne D.M., Rossomando A.J., Martino P., Erickson A.K., Her J.H., Shabanowitz J. (1991). Identification of the regulatory phosphorylation sites in pp42/mitogen-activated protein kinase (MAP kinase). EMBO J.

[29] Lisnock J., Griffin P., Calaycay J., Frantz B., Parsons J., O’Keefe S.J. (2000). Activation of JNK3 alpha 1 requires both MKK4 and MKK7: kinetic characterization of in vitro phosphorylated JNK3 alpha 1. Biochemistry.

[30] Wada T., Nakagawa K., Watanabe T., Nishitai G., Seo J., Kishimoto H. (2001). Impaired synergistic activation of stress-activated protein kinase SAPK/JNK in mouse embryonic stem cells lacking SEK1/MKK4: different contribution of SEK2/MKK7 isoforms to the synergistic activation. J Biol Chem.

[31] Geiser F. (1988). Reduction of metabolism during hibernation and daily torpor in mammals and birds: temperature effect of physiological inhibition?. J Comp Physiol B.

[32] Storey K.B., Storey J.M. (1990). Metabolic rate depression and biochemical adaptation in anaerobiosis, hibernation and estivation. Q Rev Biol.

[33] Heldmaier G., Steiger R., Ruf T., Carey C., Florant G.L., Wunder B.A., Horwitz B. (1993). Suppression of metabolic rate in hibernation. Life in the cold.

[34] Williams C.T., Goropashnaya A.V., Buck C.L., Fedorov V.B., Kohl F., Lee T.N. (2011). Hibernating above the permafrost: effects of ambient temperature and season on expression of metabolic genes in liver and brown adipose tissue of arctic ground squirrels. J Exp Biol.

[35] Maistrovski Y., Biggar K.K., Storey K.B. (2012). HIF-1alpha regulation in mammalian hibernators: role for non-coding RNA in HIF-1alpha control during torpor in ground squirrels and bats. J Comp Physiol B.

[36] Storey K.B. (2002). Life in the slow lane: molecular mechanisms of estivation. Comp Biochem Physiol A.

[37] Shaul Y.D., Seger R. (2007). The MEK/ERK cascade: from signaling specificity to diverse functions. Biochim Biophys Acta.

[38] Felton-Edkins Z.A., Fairley J.A., Graham E.L., Johnston I.M., White R.J., Scott P.H. (2003). The mitogen-activated protein (MAP) kinase ERK induces tRNA synthesis by phosphorylating TFIIIB. EMBO J.

[39] Wu C.W., Biggar K.K., Storey K.B. (2014). Expression profiling and structural characterization of microRNAs in adipose tissues of hibernating ground squirrels. Genomics Proteomics Bioinformatics.

[40] Dark J. (2005). Annual lipid cycles in hibernators: integration of physiology and behavior. Annu Rev Nutr.

[41] Brooks S.P.J., Storey K.B. (1992). Mechanisms of glycolytic control during hibernation in the ground squirrel *Spermophilus lateralis*. J Comp Physiol B.

[42] Cargnello M., Roux P.P. (2011). Activation and function of the MAPKs and their substrates, the MAPK-activated protein kinases. Microbiol Mol Bio Rev.

[43] Milne D.M., Campbell L.E., Campbell D.G., Meek D.W. (1995). P53 phosphorylated *in vitro* and i*n vivo* by an ultraviolet radiation-induced protein kinase characteristic of the c-Jun kinase, JNK1. J Biol Chem.

[44] Shi Y., Nikulenkov F., Zawacka-Pankau J., Li H., Gabdoulline R., Xu J. (2014). ROS-dependent activation of JNK converts p53 into an efficient inhibitor of oncogenes leading to robust apoptosis. Cell Death Differ.

[45] Zarubin T., Han J. (2005). Activation and signaling of the p38 MAP kinase pathway. Cell Res.

[46] Storey K.B., Storey J.M. (2011). Heat shock proteins and hypometabolism: adaptive strategy for proteome preservation. Res Rep Biol.

[47] Tibbetts R.S., Brumbaugh K.M., Williams J.M., Sarkaria J.N., Cliby W.A., Shieh S.Y. (1999). A role for ATR in the DNA damage-induced phosphorylation of p53. Genes Dev.

[48] Banin S., Moyal L., Shieh S., Taya Y., Anderson C.W., Chessa L. (1998). Enhanced phosphorylation of p53 by ATM in response to DNA damage. Science.

[49] Larsen J.K., Yamboliev I.A., Weber L.A., Gerthoffer W.T. (1997). Phosphorylation of the 27-kDa heat shock protein via p38 MAP kinase and MAPKAP kinase in smooth muscle. Am J Physiol.

[50] Maizels E.T., Peters C.A., Kline M., Cutler R.E., Shanmugam M., Hunzicker-Dunn M. (1998). Heat-shock protein-25/27 phosphorylation by the delta isoform of protein kinase C. Biochem J.

[51] Kang S., Elf S., Lythgoe K., Hitosugi T., Taunton J., Xiong L. (2010). P90 ribosomal S6 kinase 2 promotes invasion and metastasis of human head and neck squamous cell carcinoma cells. J Clin Invest.

[52] Frank C.L. (1993). The influence of dietary fatty acids on hibernation by golden-mantled ground squirrels (*Spermophilus lateralis*). Physiol Zool.

[53] Geiser F., Kenagy G.J. (1993). Dietary fats and torpor patterns in hibernating ground squirrels. Can J Zool.

[54] Génin F., Perret M. (2003). Daily hypothermia in captive grey mouse lemurs (*Microcebus murinus*): effects of photoperiod and food restriction. Comp Biochem Physiol B.

